# Eco‐friendly microwave‐assisted extraction of fruit and vegetable peels demonstrates great biofunctional properties

**DOI:** 10.1002/fsn3.4463

**Published:** 2024-09-12

**Authors:** Hülya Şen Arslan

**Affiliations:** ^1^ Department of Food Engineering Karamanoğlu Mehmetbey University Karaman Turkey

**Keywords:** anticancer, antidiabetic, antimicrobial, antioxidant

## Abstract

This study aimed to determine the biofunctional properties, such as antioxidant, antimicrobial, anticancer, and antidiabetic activities, of peel extracts obtained through microwave‐assisted extraction (MAE) of red beet, dragon fruit, and prickly pear peels using water as a green solvent. Results indicated that the peel extracts exhibited high total phenolic content (TPC), ranging from 345.93 to 1651.17 mg GAE/L. The DPPH scavenging capacity of the extracts ranged between 7.16 and 34.65 mg/mL, while the ABTS scavenging capacity ranged between 0.93 and 15.27 mg/mL. Dragon fruit peel extract (DFE) and prickly pear peel extract (PPE) showed significant α‐glucosidase inhibition effects, with 67.79% and 37.50% inhibitions, respectively. Moreover, significant antibacterial activities were observed against five pathogenic bacterial strains (*B. cereus*, *E. coli*, *S. aureus*, *L. monocytogenes*, and *S. enterica*) at various concentrations of extracts. The cytotoxic effect of the extracts on HT‐29 and HeLa cancer cells was also observed. The most abundant phenolic compound in DFE was rutin (0.558 mg/g); in PPE, hesperidin was the most abundant (0.596 mg/g); and in red beet peel extract (RBE), hesperidin (0.426 mg/g) was the predominant compound.

## Graphical abstract

Waste utilization or waste valorization. Microwave‐assisted extraction increased bioactive properties of peel extracts.

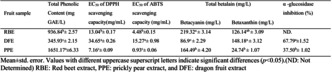

## 1 INTRODUCTION

In order to implement sustainable food production and environmentally friendly approaches, studies are carried out on reusing agricultural industrial wastes and presenting them for consumption. In particular, the reuse of food by‐products is of great importance in terms of reducing waste. Generally, fruit and vegetable byproducts such as peels, seeds, and pulp are wasted. Agricultural and industrial wastes are diverted to the food industry for a wide range of products such as stems, leaves, roots, and barks, which contain appreciable amounts of valuable nutrients and bioactive compounds (Sadh et al., 2018). Waste materials, specifically peels and seeds obtained from fruit and vegetable processing processes, contain high amounts of phenolic compounds and are considered as a rich source of phytochemicals and antioxidants. Moreover, the reuse of plant by‐products involves the process of creating new resources commercially, as well as reducing environmental pollution (Ali et al., 2022; John et al., 2017; Lazăr et al., 2022). Byproducts such as peels and seeds from fruit and vegetable processing plants have beneficial effects on the sustainable economy and environment. Moreover, plant by‐products can also be used as a natural additive to improve the quality of food products (El‐Beltagi et al., 2022; John et al., 2017; Lazăr et al., 2022).

Phytochemicals present in fruits and vegetables have diverse functions, particularly with the antioxidant activity of their phenolic compounds. Red beet, dragon fruit, and prickly pear are known for their abundance in functional properties and richness in bioactive components (Amorim et al., 2023; Shah et al., 2023). Betacyanins, a type of betalain pigment found in these fruit peels, give them a red‐violet hue. Research suggests that betacyanins possess three times more potent antioxidant properties compared to the red‐purple‐blue dyes produced by anthocyanins (Kumar et al., 2023). Dietary betalains play a crucial role in preserving human health due to their numerous beneficial activities. There are ample data available on the health effects of betalains. However, in many studies, researchers use plant extracts or other preparations that contain not only betalains but also numerous other bioactive compounds. Therefore, it is important to evaluate not only betalains but also phenolic compounds because phenolic compounds are known for their antimicrobial and antioxidant activities, which contribute to improving human health (Sadowska‐Bartosz & Bartosz, 2021).

The techniques used for preparing and extracting phytochemicals from plant materials, as well as studying their antioxidant activity and application in food products, are critical processes. However, many extraction methods employed in the food industry involve the use of chemical solvents and high temperatures (Alsobh et al., 2024). High temperatures may potentially degrade heat‐sensitive bioactive compounds present in the plant materials, and a substantial amount of solvent is often necessary to achieve maximum extraction of the desired compound (Zhou et al., 2024). Various methods, including solvent extraction, ultrasound‐assisted extraction, and soxhlet extraction, have been explored for extracting betalain from different materials. However, these traditional methods often suffer from drawbacks such as low efficiency, complexity, lengthy extraction times, and high costs (Şen Arslan & Saricoban, 2023). Microwave‐assisted extraction (MAE) has emerged as a promising alternative, offering several advantages such as high reproducibility, shorter extraction durations, simplified operation, reduced solvent consumption, and lower energy requirements, all while maintaining high extraction yields (Yildiz et al., 2024). Due to these benefits, MAE is increasingly being adopted for extracting bioactive components from food waste resources.

This study aimed to investigate an eco‐friendly method of MAE of red beet, dragon fruit, and prickly pear peels using water as a green solvent. Moreover, the biofunctional properties such as antioxidant, antimicrobial, anticancer, and antidiabetic activities of these extracts were finally evaluated in order to evaluate their potential as food additives and pharmaceutical products.

## 2 MATERIALS AND METHODS

### 2.1 Materials and chemicals

All chemical compounds used in the analysis were of high analytical grade and purchased from Sigma‐Aldrich, Turkey. Red beet, prickly pear, and dragon fruit were purchased from local markets in Karaman and brought to the laboratory. The materials were washed to remove coarse dirt upon arrival at the laboratory. The skins of the materials were then peeled, and the peels were frozen at −18°C for 2 h. Subsequently, the frozen samples were subjected to freeze‐drying in a lyophilizer (ScanvacCoolSafe 4–15 L Freeze Dryer 95/55–80, Lynge, Denmark) at −101°C for 2 days to reach a moisture content of 3%. Once dried, the samples were ground into powder prior to the extraction process.

### 2.2 Microwave‐assisted water extraction

The extraction was conducted at a fixed value of 800 W‐150 s, which was taken as an optimum point from a previous study on MAE of dried peels (Zin & Bánvölgyi, 2023). Extracts were prepared using ultrapure water as the solvent, with a solid‐water ratio of 1:10 (w/v). The mixture was centrifuged at 1860*g* for 10 min and then filtered separately through coarse filter paper. Subsequently, the filtered red beet extract (RBE), prickly pear extract (PPE), and dragon fruit extract (DFE) were stored in small (10 mL) sterile bottles at −18°C until in vitro biological assays.

### 2.3 Analysis of the extracts

#### 2.3.1 Total phenolic content

Total phenolic content (TPC) of the extracts was determined according to the method of Cam and Icyer (2015). TPC values were expressed as mg gallic acid equivalent (GAE)/L.

#### 2.3.2 Antioxidant capacity by DPPH and ABTS methods

Two in vitro antioxidant assays, ABTS and DPPH radical scavenging activities, were carried out with the extracts. The details of method protocols of ABTS can be found in the study of Fan et al. (2015) and the DPPH method in the work of Fernandes et al. (2016). Final results were expressed as EC_50_ values.

#### 2.3.3 Total betalain content

Betacyanins and betaxanthins content of the extracts were determined spectrophotometrically following the Nilsson's method (Koubaier et al., 2014).

#### 2.3.4 Alfa‐glucosidase inhibitory activity

The activity of α‐glucosidase was determined by colorimetric methods (McDougall et al., 2005; Şen Arslan & Çam, 2022).

#### 2.3.5 Antibacterial test

The microdilution water method was used to determine the MIC (minimum inhibitor concentration) of extracts. *B. cereus*, *E. coli*, *S. aureus*, *L. monocytogenes*, and *S. enterica* cultures were tuned to 10^6^ colony‐forming units (CFU/mL) overnight (Bayat et al., 2023).

#### 2.3.6 Cell experiment

The cytotoxicity in epithelial cells (L9‐29) and cancer cell lines (HT‐29 and HeLa) was evaluated using the MTT test (Yilmaz, 2022; Yilmaz et al., 2020).

#### 2.3.7 Phenolic fractions

The phenolic profile analysis was conducted following the conditions outlined by Angonese et al. (2021). MS detection was performed using a Shimadzu LCMS‐8030 model triple quadrupole tandem mass spectrometer equipped with an electrospray ionization source.

### 2.4 Statistical analysis

The analyses were performed with two repetitions and three replicates, resulting in 6 independent measurements. Statistical analysis was carried out using the one‐way ANOVA model in the SPSS 22 statistical package for Windows (IBM Corp., Armonk, New York, USA), followed by Tukey's pairwise comparison of the means, with a significance level set at 5%.

## 3 RESULTS AND DISCUSSION

### 3.1 Characterization and α‐glucosidase inhibition of extracts

The characterization of the extracts was examined in terms of total phenolic content, antioxidant activity, and total betalain. The total phenolic content (TPC) of extracts was analyzed with the Folin–Ciocalteu method and presented in Table 1. TPC contents ranged from 345.93 to 1651.17 mg GAE/L. The highest TPC was found in PPE. On the other hand, DFE showed the lowest TPC, which was ~4.5 fold lower than PPE. Given that various compounds within fruits and vegetables exhibit antioxidant properties, and the effectiveness of these properties varies depending on the specific nature of each compound, a singular method is insufficient for assessing the antioxidant potential of a given fruit or vegetable (Arivalagan et al., 2018). Therefore, in this study, we evaluated the antioxidant potential of peels using both radical scavenging assays, including DPPH and ABTS activities. The results of the antioxidant activity of the extracts were expressed using the term EC_50_. All of the extracts were found to have high antioxidant activity. The DPPH scavenging capacity of the extracts ranged between 7.16 and 34.65 mg/mL, while the ABTS scavenging capacity ranged between 0.93 and 15.27 mg/mL (Table 1). The ABTS radical scavenging potential exhibited notably higher activity compared to the DPPH radical scavenging activity. This difference can be attributed to the fact that ABTS radicals have the ability to interact with both hydrophilic and lipophilic antioxidants, as ABTS is soluble in both aqueous and organic solvent systems. In contrast, DPPH is soluble solely in organic mediums (Arivalagan et al., 2018; Bibi Sadeer et al., 2020). PPE, which exhibited the highest value in terms of TPC, also demonstrated the highest activity in terms of antioxidant activity. It is known that radical scavenging activity is typically related to the TPC. Accordingly, in this study, antioxidant activities were found to be comparable to TPC. Prickly pear possesses defensive properties due to the presence of antioxidant compounds such as vitamin C, vitamin E, betalains, polyphenols like flavonoids, and phenolic acids (Sabtain et al., 2021).

**TABLE 1:** TPC, BLC, antioxidant, and enzyme inhibition activity of extracts.

Fruit sample	Total phenolic content (mg GAE/L)	EC_50_ of DPPH scavenging capacity (mg/mL)	EC_50_ of ABTS scavenging capacity (mg/mL)	Total betalain (mg/L)		α‐Glucosidase inhibition (%)
				Betacyanin (mg/L)	Betaxanthin (mg/L)	
RBE	936.84^b^ ± 2.57	13.04^b^ ± 0.17	4.48^b^ ± 0.15	219.32^a^ ± 3.14	126.14^ab^ ± 3.09	ND
DFE	345.93^c^ ± 2.15	34.65^a^ ± 0.26	15.27^a^ ± 0.98	86.9^c^ ± 2.29	148.18^a^ ± 3.12	67.79^a^ ± 1.52
PPE	1651.17^a^ ± 6.33	7.16^c^ ± 0.09	0.93^c^ ± 0.06	164.49^b^ ± 4.20	24.74^b^ ± 1.07	37.50^b^ ± 1.02

*Note*: Mean ± std. error. Values with different lowercase superscript letters indicate significant differences (*p* < .05).

Abbreviations: DFE, dragon fruit extract; ND, not determined; PPE, prickly pear extract; RBE, red beet extract.

RBE, PPE, and DFE contain a group of water‐soluble pigments known as betalains, which are found in both the peel and flesh. These pigments primarily include betacyanin and betaxanthin (Araujo et al., 2022). Total betalein concentrations (BLC) of RBE, DFE, and PPE were 345.46 mg/L, 235.08 mg/L, and 189.23 mg/L, respectively. The most renowned edible sources of betalains include red beet roots, grainy or leafy amaranth, the prickly pear, and dragon fruit (Sadowska‐Bartosz & Bartosz, 2021). The concentration of these pigments is highest on the surface (peel) of the fruit or vegetable and gradually decreases toward the interior (Sadowska‐Bartosz & Bartosz, 2021).

Type 2 diabetes is characterized by postprandial hyperglycemia and inadequate insulin secretion. Managing type 2 diabetes often involves delaying glucose absorption, achieved through the inhibition of carbohydrase enzymes such as α‐glucosidase and α‐amylase (Girish et al., 2012). It has been reported that polyphenols derived from plants are effective inhibitors of these enzymes (Zahid et al., 2022). Table 1 presents the inhibitory potential of extracts on α‐glucosidase. The antidiabetic potential of peel extracts was assessed through in vitro α‐glucosidase inhibition tests. The results indicated that DFE and PPE exhibited significant effects in the α‐glucosidase inhibition effects, with 67.79% and 37.50% inhibitions, respectively. Sarker et al. (2023) found that the α‐glucosidase enzyme inhibition of extracts obtained from dragon fruit peel and fruit using the classical method with chemicals was 56.42% and 46.63%, respectively. Younis et al. (2023) observed that the α‐glucosidase inhibition of dragon fruit extracts obtained through the classical method with methanol ranged between IC_50_ values of 1.23 and 1.53 mg/mL. Mabotja et al. (2021) observed that prickly pear extracts obtained through the classical method with methanol significantly inhibited α‐glucosidase, with IC_50_ values ranging from 0.06 to 1.85 mg/mL. α‐Glucosidase is a crucial enzyme in carbohydrate digestion, and inhibiting its activity is regarded as a therapeutic strategy for controlling postprandial hyperglycemia, a prevalent issue in type‐2 diabetes. α‐Glucosidase inhibitors can effectively manage hyperglycemia by postponing the onset of postprandial hyperglycemia (Şen Arslan & Çam, 2022).

### 3.2 Antibacterial activity of extracts

In this study, antibacterial activity was assessed using the microdilution water method with *B. cereus*, *E. coli*, *S. aureus*, *L. monocytogenes*, and *S. enterica*. The minimum inhibitory concentration (MIC), representing the lowest concentration of peel extracts preventing visible bacterial growth, was determined. MIC values for the three different extracts ranged from 0 to 50 mg/mL (Table 2). The most effective MIC was observed with PPE at 20 mg/mL against *S. aureus*, as this concentration completely inhibited bacterial growth. The least efficacy was demonstrated by RBE against *S. aureus*, with inhibition observed at a concentration of 20 mg/mL.

**TABLE 2:** Minimum inhibitory concentration of extracts.

Fruit sample	Minimum inhibitory concentration (mg/mL)								
	Percent of bacterial colonies survived at above concentration								
		0	1	2	5	10	15	20	50
RBE	*B. cereus* (+Ve)	99.70 ± 3.36	94.01 ± 4.55	75.93 ± 3.99	71.23 ± 2.36	48.59 ± 3.00	42.84 ± 4.16	22.75 ± 1.22	0
	*E. coli* (−Ve)	99.29 ± 3.25	95.61 ± 3.49	75.92 ± 2.36	55.41 ± 2.25	12.05 ± 1.12	11.33 ± 1.23	0	0
	*S. aureus* (+Ve)	98.51 ± 4.58	91.90 ± 6.45	91.42 ± 4.48	89.44 ± 4.45	85.72 ± 4.45	85.34 ± 3.36	47.84 ± 1.21	0
	*L. monocytogenes* (+Ve)	99.46 ± 5.54	98.92 ± 5.45	97.24 ± 5.56	86.89 ± 4.65	74.27 ± 2.25	47.92 ± 2.23	37.99 ± 1.26	0
	*S. enterica* (−Ve)	99.90 ± 6.25	97.32 ± 3.36	91.02 ± 4.52	86.53 ± 4.48	79.85 ± 2.29	23.79 ± 1.26	0	0
DFE	*B. cereus* (+Ve)	99.70 ± 3.36	87.16 ± 3.67	70.15 ± 2.26	16.29 ± 1.15	15.06 ± 1.15	12.71 ± 1.02	0	0
	*E. coli* (−Ve)	99.29 ± 3.25	97.50 ± 5.45	88.80 ± 4.42	80.99 ± 8.54	15.78 ± 1.12	13.77 ± 1.25	10.57 ± 1.12	0
	*S. aureus* (+Ve)	98.51 ± 4.58	93.71 ± 4.48	92.59 ± 5.51	78.47 ± 3.26	63.83 ± 2.15	45.77 ± 3.21	10.29 ± 1.13	0
	*L. monocytogenes* (+Ve)	99.46 ± 5.54	98.92 ± 5.89	97.24 ± 4.20	86.89 ± 3.54	74.27 ± 3.25	47.92 ± 3.36	37.94 ± 1.26	0
	*S. enterica* (−Ve)	99.90 ± 6.25	85.18 ± 3.96	83.92 ± 3.15	81.32 ± 3.33	11.01 ± 1.12	10.61 ± 1.21	0	0
PPE	*B. cereus* (+Ve)	99.70 ± 3.36	91.47 ± 3.45	77.23 ± 4.45	71.71 ± 2.26	63.71 ± 2.23	11.83 ± 1.36	0	0
	*E. coli* (−Ve)	99.29 ± 3.25	98.90 ± 6.15	98.62 ± 6.65	79.29 ± 3.36	13.66 ± 1.26	13.45 ± 1.25	10.72 ± 1.56	0
	*S. aureus* (+Ve)	98.51 ± 4.58	87.05 ± 2.25	52.89 ± 5.54	9.55 ± 096	8.57 ± 1.02	0	0	0
	*L. monocytogenes* (+Ve)	99.46 ± 5.54	98.92 ± 6.85	97.24 ± 4.45	86.89 ± 5.56	74.27 ± 3.45	47.92 ± 3.26	37.94 ± 2.23	0
	*S. enterica* (−Ve)	99.90 ± 6.25	84.23 ± 7.48	78.95 ± 5.56	73.19 ± 4.41	12.38 ± 1.20	12.04 ± 1.23	0	0

*Note*: Values were represented as a means ± standard deviation of six data.

Abbreviations: DFE, dragon fruit extract; PPE, prickly pear extract; RBE, red beet extract.

The addition of different concentrations of extracts to the bacterial growth medium led to a reduction in bacterial growth. With increasing concentrations, the growth of *B. cereus, E. coli, S. aureus, L. monocytogenes*, and *S. enterica* bacteria declined. When a 20 mg/mL extract was used, RBE completely inhibited *E. coli* and *S. enterica*; DFE completely inhibited *B. cereus* and *S. enterica*; and PPE inhibited *B. cereus*, *S. aureus*, and *S. enterica*. The extracts caused a decrease in the growth rate of both Gram‐negative and Gram‐positive bacteria. It was observed that extracts had varying effects on different bacterial species.

Betalains and phenolics have demonstrated antibacterial properties. Extracts containing betalains from prickly pear have been shown to inhibit the growth of *E. coli* (Madadi et al., 2020). Likewise, beetroot pomace has been reported to inhibit the proliferation of *S. typhimurium*, *S. aureus*, and *B. cereus* (de Oliveira Filho et al., 2022). In another study, beetroot pomace exhibited inhibitory effects on Gram‐negative bacteria (Amin et al., 2024). Extracts rich in betalains from dragon fruit inhibited Gram‐positive bacteria and Gram‐negative bacteria (Carmen et al., 2023). The antimicrobial activity of betalains and phenolics likely involves their effects on the structure, permeability, and other functions of microbial cellular membranes, ultimately resulting in cell death (Şen Arslan, 2023).

### 3.3 Anticancer activity of extracts

**FIGURE 1:**
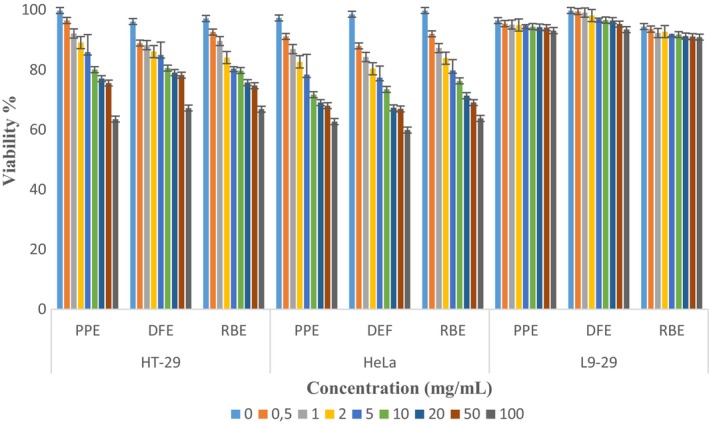
In vitro anticancer results of extracts on different cell lines. Values were represented as a means ± standard deviation of six data. DFE, dragon fruit extract; PPE, prickly pear extract; RBE, red beet extract.

After completing the characterization of extracts obtained via MAE, cell cytotoxicity tests were conducted initially. The cytotoxicity of extracts on epithelial (L9‐29) and cancer cell lines (HT‐29 and HeLa) was examined using the MTT® assay. The viability results obtained from the cytotoxicity tests are presented in Figure 1. As a result of the study conducted at various concentrations (0–100 mg/mL), it was observed that the viability (%) decreased with increasing extract concentrations. For PPE, DFE, and RBE, the viability (%) in the HT‐29 cell line was found to be 63.54, 67.22, and 66.89, respectively, while in the HeLa cell line, it was found to be 62.72, 59.89, and 63.78, respectively. Specifically, extracts that exhibited high cytotoxicity against cancer cell lines showed lower cytotoxicity in epithelial cells. According to the cytotoxicity results, it was observed that the addition of extracts had a strong anticarcinogenic effect on cancer cells.

Numerous experiments have shown that betalains and phenolic plant extracts exhibit cytotoxic effects on cancer cell lines. While betalains are generally considered non‐toxic up to concentrations of 100 μM and are safe for non‐cancerous cell cultures, some cell cultures may experience growth inhibition in the presence of extracts (de Freitas Marinho et al., 2023; Kumari et al., 2024; Thiruvengadam et al., 2024; Zand et al., 2024). Betalain isolated from prickly pear induced apoptosis in human chronic myeloid leukemia cells, and red beet extracts showed limited cytotoxicity against human breast cancer and prostate cancer cell lines (Thiruvengadam et al., 2024). Extracts from prickly pear inhibited growth and induced apoptosis in various ovarian, cervical, and bladder cancer cell lines, as well as in immortalized ovarian and cervical epithelial cells. Similarly, extracts from elder fruit inhibited the growth of melanoma cells, with peel extracts exhibiting stronger effects due to their higher content of betalains and flavonoids (Martins et al., 2023).

### 3.4 Phenolic compounds profile comparison of extracts

**FIGURE 2:**
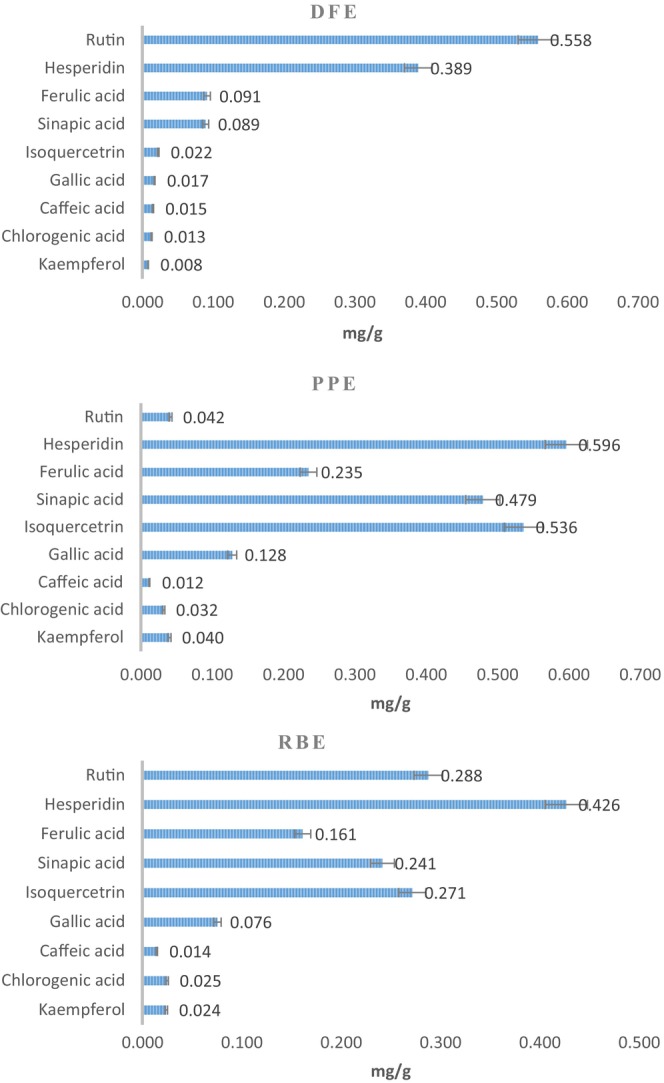
Phenolic compound profiles of extracts. Values were represented as a means ± standard deviation of six data. DFE, dragon fruit extract; PPE, prickly pear extract; RBE, red beet extract.

The phenolic compositions of DFE, PPE, and RBE are provided in Figure 2. LC/MS–MS analyses enabled the identification of 9 phenolic compounds in peel extracts. The concentrations of these compounds varied widely, ranging from 0.008 to 0.596 mg/g. The most abundant phenolic compound in DFE was rutin (0.558 mg/g), followed by hesperidin (0.389 mg/g) and ferulic acid (0.091 mg/g). In PPE, hesperidin was the most abundant (0.596 mg/g), followed by isoquercetrin (0.536 mg/g) and sinapic acid (0.479 mg/g). In RBE, hesperidin (0.426 mg/g) was the predominant compound, followed by rutin (0.288 mg/g) and isoquercetrin (0.271 mg/g).

Rutin and hesperidin are flavonoid compounds known for their functional and bioactive properties, commonly found in fruits and vegetables (Du et al., 2018; Patel & Patel, 2019). Rutin has also been identified as an important compound in seeds and peel for both red beet and dragon fruit (Angonese et al., 2021; Ben Haj Koubaier et al., 2014; Nguyen et al., 2019). Additionally, rutin is frequently found in berries (Diaconeasa et al., 2014), red‐purple colored fruits, and grapes (Li et al., 2019), as well as in plant‐derived beverages such as wine and teas (Elçin et al., 2016). Mena et al. (2018) identified 26 phenolic compounds from prickly pear peel and 21 compounds from its pulp, with 9 phenolic compounds overlapping with those identified in this study. It is understood that the concentration of (poly)phenolic compounds in fruits and vegetables varies depending on genetic factors, as well as extraction conditions and the solvent used (Khatabi et al., 2016; Moussa‐Ayoub et al., 2014). Previous studies have investigated the (poly)phenolic composition of various parts of fruits and vegetables (Moussa‐Ayoub et al., 2014; Yeddes et al., 2014).

## 4 CONCLUSION

This study well demonstrated that MAE of fruit peels using water exhibits antioxidant, antimicrobial, anticancer, and antidiabetic activities. The highest total phenolic content (TPC) and antioxidant activity were observed in PPE, while the highest betalain content (BLC) was found in RBE, and the highest α‐glucosidase inhibition activity was observed in DFE. Significant antibacterial activities were observed at various concentrations of extracts against five pathogenic bacterial strains (*B. cereus, E. coli, S. aureus, L. monocytogenes*, and *S. enterica*). Subsequently, the results of cytotoxicity experiments strongly suggest that peel extracts are safe additives and ingredients in the food and pharmaceutical industries. Finally, the MAE process revealed that the peels, previously considered as waste, possess functional properties due to their high phenolic and betalain content.

## CONFLICT OF INTEREST STATEMENT

The author confirms that there are no known conflicts of interest associated with this study.

## Data Availability

No data available.
